# Assessment of the properties of concrete containing artificial green geopolymer aggregates by cold bonding pelletization process

**DOI:** 10.1007/s11356-024-32987-7

**Published:** 2024-03-21

**Authors:** Taher A. Tawfik, Alaa Hussein Kamal, Ahmed Serag Faried

**Affiliations:** 1 Institute of Construction and Architecture, Slovak Academy of Sciences, Dúbravskácesta 9, SK-845 03 Bratislava, Slovak Republic; 2 Department of Construction and Building Engineering, High Institute of Engineering, October 6 City, Egypt; 3 Faculty of Engineering, Fayoum University, Fayoum, Egypt; 4 Civil Engineering Department, Faculty of Engineering, Fayoum University, Fayoum, Egypt

**Keywords:** Artificial green geopolymer coarse aggregates, Cold-bonded pelletization, GGBFS, Fly ash, Perlite, Physical and mechanical characteristics

## Abstract

Recently, the usage of a cold-bonded method in the production of artificial green geopolymer coarse aggregates (GCA) has been crucial from an economic and environmental perspective because the sintering method consumes an enormous quantity of energy and generates a significant quantity of pollutants. This research investigated the manufacture of GCA via cold-bonded pelletization using two distinct industrial byproducts (GGBFS and FA) via a new and simpler pelletization technology. Three different binders were used to produce three distinct types of GCAs as partial replacements for natural coarse aggregate (NCA) at varying replacement rates (0%, 25%, 50%, and 75%). The first group used ground-granulated blast furnace slag, while the second used GGBFS with perlite, and the third used FA with perlite. An alkaline activator was commonly used with all three groups. The physical and mechanical properties of three distinct varieties of GCA were recorded. The results indicated that the mechanical and chemical properties of three different types of GCAs were nearly identical to those of natural aggregate, with the exception of their increased water absorption. According to the findings, the recommended mixtures were suitable for usage in the construction industry. The results indicated that the ratio of all investigated attributes declined as the number of GCAs increased. In contrast, lightweight concrete can be obtained at a ratio of GCA (FA with perlite) equal to 75%, where unit weight, compressive, splitting tensile, flexural, and water absorption strengths were 1.87 gm/cm^3^, 20.2 MPa, 1.8 MPa, 8 MPa, and 6.0%, respectively (FA with perlite).

## Introduction

Concrete is a widely used structural material all over the world, known for its exceptional durability and longevity, as well as its ability to withstand varying levels of compression (Ahmed et al. 2022). Concrete’s adaptability is also one of its most important properties. Concrete is now available and practicable in any location and under any climatic conditions due to progress in the construction industry (Naik 2008). Ordinary concrete components are mainly natural resources, where aggregate is considered to be 70–80% of the concrete ingredient (Sims and Brown 1998). Sand is a fine aggregate and is considered the third-most consumed natural material after water and air (Cao et al. 2022). Coarse aggregate, such as gravel, lime, basalt, and dolomite, is a natural and limited resource that is exposed to depletion due to its many uses in the construction industry (Sims and Brown 1998). On the other hand, population growth and urbanization have increased the demand for construction materials over the past decades. Natural resources are being utilized at such a rapid rate that their long-term sustainability has become an urgent issue. On the other hand, industrial and municipal sources produce waste at an unsustainable rate for both the environment and sustainability. A viable solution for these two problems can be found in the scanty usage of resources and the recycling of waste through the manufacturing of various materials (Rehman et al. 2020). Furthermore, minerals are vital construction materials, although their utilization, handling, processing, and extraction contribute to around 7% of the overall world energy consumption. Additionally, the transportation of fundamental minerals alone accounts for 40% of the industry’s overall energy usage (Mankelow et al. 2010).

In terms of natural aggregate usage, the use of artificial and recycled aggregates as a partial replacement for natural aggregates must be encouraged. In this context, massive volumes of industrial solid wastes, sludges, reservoir sediments, and demolition wastes are recycled in the manufacturing of both concrete and aggregates, which is considered a critical problem in driving waste management toward a more sustainable concrete construction future besides more long-term development (Colangelo and Cioffi 2013; Naik 2008). The cement industry uses raw materials as the base of the manufacturing process and also emits a large amount of harmful CO_2_ gas into the air, which affects the environment badly and causes global warming (Ali et al. 2015). Eco-friendly industries and recycling waste materials are also some of the most difficult challenges faced nowadays because of the multi-production industries (Nandy et al. 2022). Given the aforementioned problems, the conversion of unused waste materials into artificial aggregates for use in concrete has emerged as a significant subject of interest. Nowadays, the main procedures used for producing artificial aggregates are cold bonding and sintering (Bijen 1986). The cold bonding method commonly employs cementitious materials, such as cement or lime, to bind waste products, including fly ash (FA), furnace bottom ash, and ground-granulated blast furnace slag (GGBFS). This binding process occurs at curing temperatures below 100 °C (Aljerf 2015). This method, which is employed on dry powder fly ash, is known as the cold bonding palletization procedure. Fly ash pellets are created by agglomerating fly ash particles in a tilted rotating pan at room temperature, with water acting as a wetting agent and Portland cement and/or lime acting as a binder. This approach consumes much less energy for producing artificial aggregate than autoclaving or sintering (İpek et al. 2020). Agglomeration is the first step in the production of cold-bonded aggregates (CBA), which is accomplished by the use of waste and side-effect fine materials. When fines are overhauled into larger particles, these are either by the use of pressure or non-pressure agglomeration procedures (Sivakumar and Gomathi 2012). The production process is affected by many parameters; the use of low angles and high velocities in pelletizers reduces the growth paths of particles, which causes them to collide with each other, resulting in the formation of small aggregates with low efficiency (Manikandan and Ramamurthy 2009). Many papers in cold-bonded aggregate production use cement as a binder to get fresh pellets, as reviewed by Tajra et al. (2019), who also summarized the benefits of cold-bonded aggregate.

Alkali-activated materials have undergone significant advancements in recent decades (Alrefaei et al. 2019; Wang et al. 2021; Thang 2020; Minh and Thang 2020), giving rise to a novel category of aggregates known as alkali-activated aggregates. Among these aggregates are cold-bonded aggregates, which form the primary focus of this article. Geopolymer materials are created by incorporating alkali activators into industrial aluminosilicate waste materials such as fly ash (FA), ground-granulated blast furnace slag (GGBS), metakaolin (MK), and red mud (RM). Geopolymer materials derived from Si/Al-rich minerals outperform ordinary concrete in terms of mechanical and chemical qualities (Provis et al. 2015; Shilar et al. 2022). In addition, (Bui et al. 2012) conducted a paper on producing cold-bonded lightweight aggregate with an alkaline solution for high-performance concrete using different mixes of fly ash, slag, and rice husk ash. Furthermore, Geetha and Ramamurthy (2013) studied the factors and characteristics of producing cold-bonded aggregate using geopolymerized low-calcium bottom ash and an ambient temperature-curing regime and found that the aggregate’s open porosity and water absorption significantly decreased when the NaOH molarity and Na2SiO3/NaOH ratio increased. On the other hand, Risdanareni et al. (2018) mentioned that the dry density of concrete, when using alkali-activated aggregates as total or partial substitutions, varies between 1846 and 2165 kg/m^3^. These values might be further decreased by increasing the ratio of alkali-activated aggregates used as substitutions. Alkali-activated aggregates are often moistened prior to being added to concrete. This process is carried out to prevent the inaccurate water-to-cement ratio and the deficiency of concrete workability caused by the aggregates absorbing too much water (Terzic´et al. 2015; Hwang and Tran 2015; Gomathi and Sivakumar 2015; Gunasekera et al. 2018). Nevertheless, the mechanical characteristics and durability of concrete are directly influenced by the characteristics of the interfacial transition zone (ITZ), which change depending on the curing age (Scrivener et al. 2004). For example, Agrawal et al. (2019) noted that the interfacial transition zone (ITZ) of the cement matrix enclosing cold-bonded alkali-activated aggregates exhibited increased density after 90 days of curing compared to the 28th day.

The main objective of this study is to produce eco-friendly coarse aggregate from recycled waste materials such as GGBFS and fly ash with an alkaline activator[sodium silicate Na_2_SiO_3_ and 8 M of sodium hydroxide (NaOH) Na_2_SiO_3_/NaOH ratio = 2:1 with water as an alternative bond material through the pelletizing process [artificial green geopolymer coarse aggregates (GCA)]. The previous researchers used ordinary Portland cement as a binder in the cold-bonded aggregate and pelletizing disk. While this current work depends on using new techniques for the pelletizing process. In this paper, the used mixtures and new pelletizing techniques help in reducing the dependence on cement in the construction process, as cement is one of the main reasons for emitting CO_2_ into the air, and the pelletization process is easier and more applicable than before. On the other hand, the use of natural aggregate leads to high environmental costs due to the consumption of fuel during the extraction and transportation processes. Thus, the goal of research is to produce eco-friendly coarse aggregate that does not consume a high amount of fuel. Therefore, this study investigates the effects of using three different mixes of GCA as a partial replacement of natural coarse aggregate in concrete (25%, 50%, and 75%) with a new technique for the pelletization process. To demonstrate the effect of GCA on the mechanical and physical characteristics of concrete, the density, water absorption, and flexural and compressive strengths at 28 days of age were examined, and the SEM of concrete mixture samples was analyzed. Besides, environmental benefits were also evaluated.

## Materials and method

### Materials

#### Raw material

**Fig. 1:**
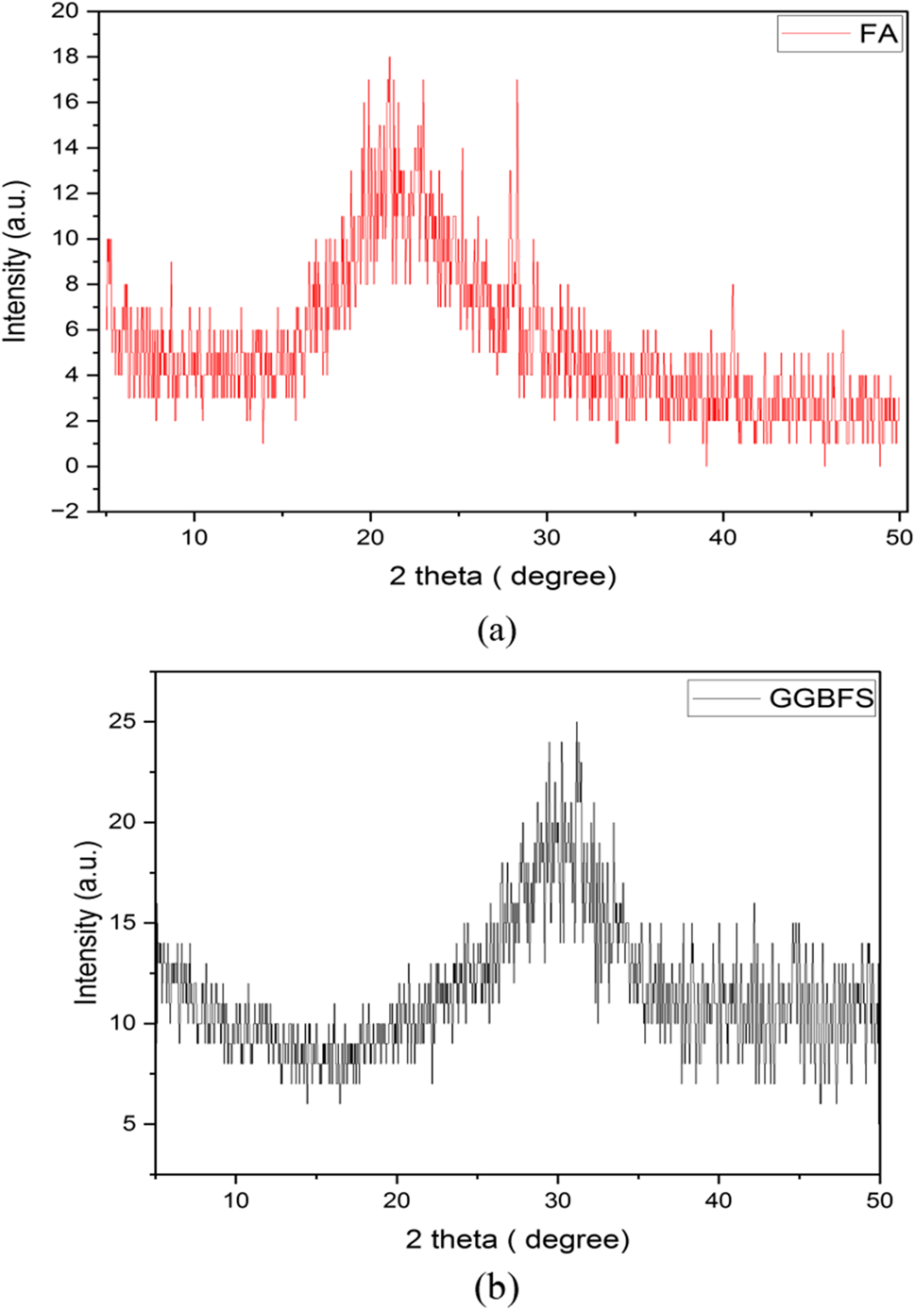
XRD pattern of used FA and GGBFS

Ordinary Portland cement (CEM I) grade 42.5 from the Bani-Swif mill was utilized in this project. It was put through a series of tests to ensure that it met Egyptian standards. Its specific gravity was 3.11, its fineness was 3400 cm^2^/gm, the initial and final setting times were 80 min and 200 min, and the compressive strengths after 3 days, 7 days, and 28 days were 196 kg/cm^2^, 290 kg/cm^2^, and 395 kg/cm^2^, respectively. The fly ash (FA) was obtained from Sika Company for chemical products. Nevertheless, the ground-granulated blast furnace slag (GGBFS) was acquired from Alexandria iron factories (Ezz steel). Figure 1 shows the XRD pattern for FA and GGBFS. Figure 1a shows that the XRD analysis of FA revealed a prominent peak corresponding to quartz with high intensity at 2theta ≈ 26.6°. This finding was supported by the XRF data, which discovered 40.20% SiO_2_ in FA. The presence of mullite was detected as the next main peak in the Fourier analysis (FA) in several 2theta ranges (16.49°, 31°, 33°, and 42°). The significant peaks seen in GGBFS (Fig. 1b) were identified as calcite at angles of 29.4°, 36°, and 39° and quartz at angles of 21.29° and 42°. These findings were supported by the XRF data, which revealed a calcium oxide content of 34.16% in GGBFS. Furthermore, the perlite powder was used as a lightweight material and was obtained from ECP on the 6th of October city. The chemical analysis of cement, fly ash, and GGBFS and the physical properties of fly ash, GGBFS, and perlite are listed in Table 1 and Table 2, respectively.

**Table 1:** The chemical analysis of materials used

Oxides	Cement	Fly ash	GGBFS
CaO	62.50	5.50	34.16
SiO_2_	20.70	40.20	20.66
FeO	____	____	0.32
Fe_2_O_3_	3.01	32.70	0,06
Fe tot	____	____	0.46
MnO	____	____	0.05
MgO	0.64	8.00	9.44
P_2_O_5_	____	____	0,01
S	____	____	1.47
Cr_2_O_3_	____	____	0.00
Al_2_O_3_	5.40	26.50	10.13
Na_2_O	0.11	34.90	____
K_2_O	____	____	____
SO_3_	2.30	____	____
C		____	1,01
F. L	2.65	____	
LOI	2.27	____	____

**Table 2:** The physical properties of materials used

Property	Fly ash	GGBFS	Perlite
Specific gravity	2.25	2.68	2.2
Mean particle size (µm)	23.4	15.2	____
Surface area (cm^2^/g)	3110	4320	____
Bulk density (gm/cm^3^)	____	____	1.1

#### Natural aggregate

Natural aggregates were used for this investigation as fine and coarse aggregates according to ASTM C33/C33M-18: the properties of crushed dolomite (NCA) size number 1, which was employed in this study and was obtained from the El Minya quarry. To guarantee that all dust was eliminated from coarse aggregates, water was used to wash them before use. In addition, the fine aggregate passed through 4.75 mm. Sieve sand from the Helwan quarry was utilized as a fine aggregate. It was pure and free of pollutants as well as biological matter. The properties of the natural aggregate used in this research are listed in Table 3.

**Table 3:** The physical properties of natural aggregate

Property	Natural sand	Crushed dolomite
Specific gravity	2.52	2.65
Fineness modulus	2.4	2.98
Bulk density (t/m^3^)	1.75	1.55
Crushing strength (%)	**____**	19.3
Absorption (%)	1.7	1.9
Total moisture content (%)	0.1	0.5

#### Green geopolymer coarse aggregate production

The current investigation introduced a method to manufacture artificial green geopolymer coarse aggregate (GCA). Experimental tests were carried out to ensure the properties of GCA. First of all, manufacturing GCA by using pelletization discs was a bit difficult, so in the present work, a new technique was used as the new technique made it easier to produce the artificial aggregate with a mini mixer, which is a tool that already existed in the lab. In addition, green GCA was prepared by mixing GGBFS with an alkaline solution in the first group. Nevertheless, in the second group, GGBFS was used with perlite and was mixed with fly ash with perlite in the third group. Table 4 provides the proportions for three different types of green GCA. The mix proportions were finalized after prior valorization during the pilot-scale study to produce good-strength aggregates. For a pelletization process technique, a mixer with 230 L was used, and the speed and the angle of the drum of the mixer were 30 rpm/min and 70°, respectively, which conform to previous works (Tajra et al. 2019; George and Revathi 2020).

**Table 4:** The content of artificial green geopolymer coarse aggregate, raw materials

Mix	GGBFS	Fly Ash	Perlite	Solution/binder
Mix (1)	3.5 kg	**____**	**____**	0.30
Mix (2)	3.5 kg	**____**	2 L	0.40
Mix (3)	____	3.5 kg	2 L	0.45

As described here, the processing procedures have a high impact on the quality of cold-bonded aggregate. Following are the steps involved in the manufacturing process of artificial green geopolymer coarse aggregate (GCA).

**Fig. 2:**
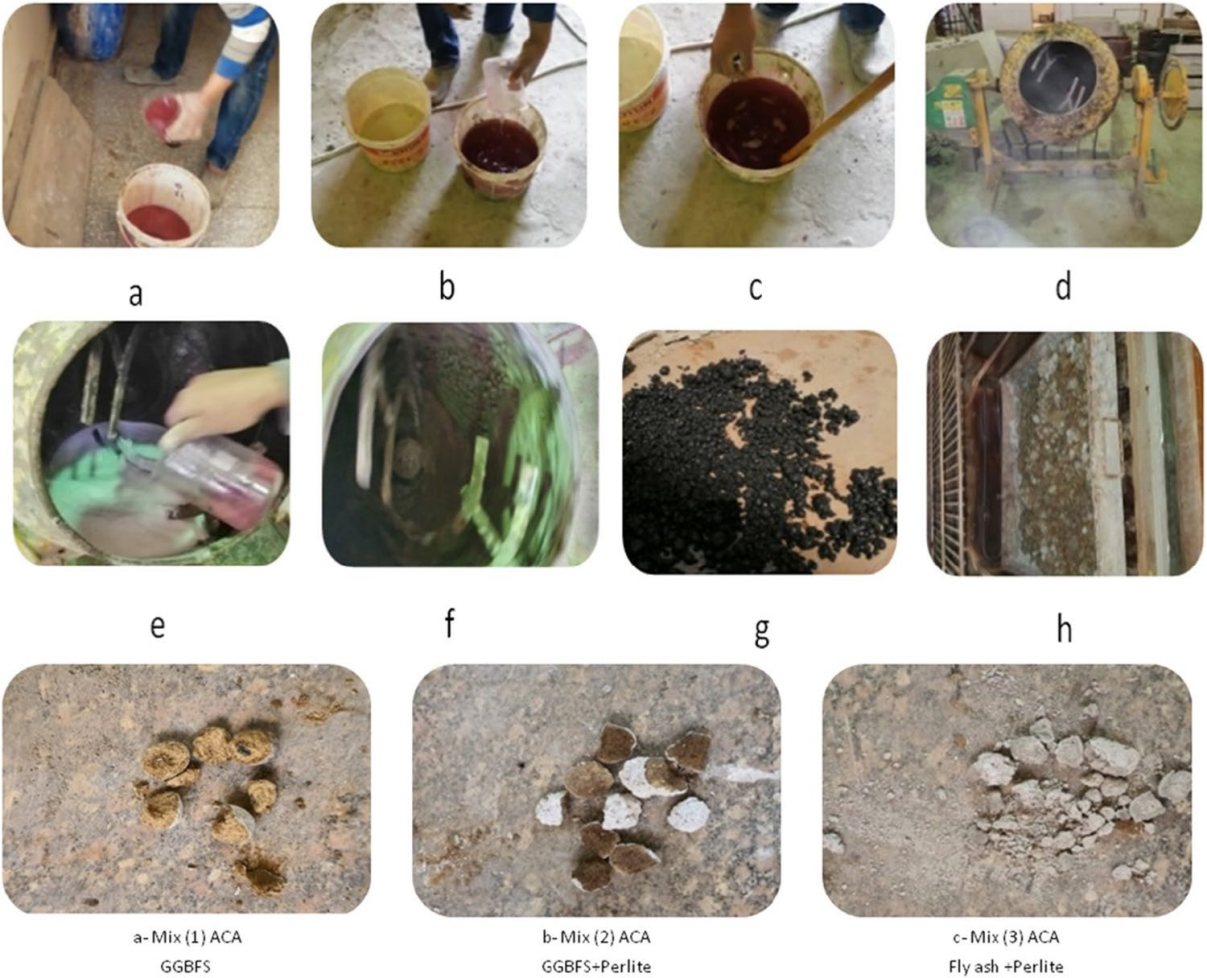
Steps of manufacturing the artificial green GCA

Firstly, the alkaline solution was prepared by mixing sodium silicate Na_2_SiO_3_ and 8 M of sodium hydroxide(NaOH) [Na_2_SiO_3_/NaOH ratio = 2:1] and letting it sit for 24 h at room temperature (Fig. 2a–c). Secondly, the lab mixer sets at 70 degrees (Fig. 2d). Thirdly, raw materials were prepared and put into the drum (Fig. 2d). Fourthly, the lab mixer was turned on for two minutes to mix the raw materials (Fig. 2d). Fifthly, the alkaline solution was added gradually (Fig. 2e). Sixthly, the mixer was set to 90 degrees and let it turn on until the fresh pellets were formed (Fig. 2f). Finally, transfer the fresh pellets from the mixer to a plate and then cured in the air for 24 h (Fig. 2g), followed by curing in the oven for 24 h at 80 °C (Fig. 2h). The fresh pellets for three different types of artificial green geopolymer coarse aggregates are displayed in Fig. (2i).

#### Test procedures on artificial green GCA

**Fig. 3:**
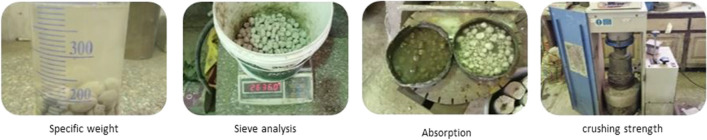
Photographs of several tests conducted on artificial green GCA

**Fig. 4:**
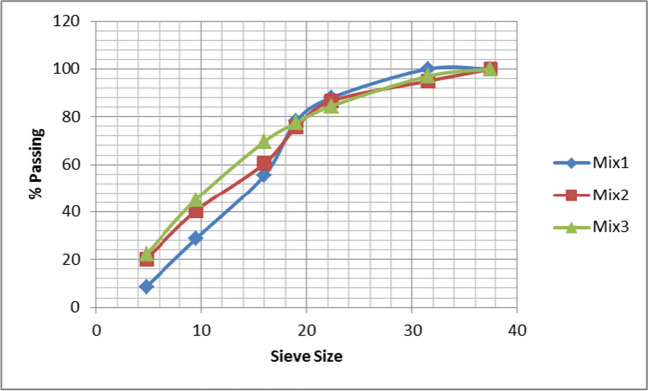
Gradation curves of the artificial green GCA

After curing the three types of artificial green geopolymer coarse aggregate (GCA), some tests were conducted according to ASTM C128 and ASTM C127 in order to ensure the quality of the produced artificial green GCA as a mechanical and chemical properties such as granular gradient, specific gravity, volume weight, crushing strength, water absorption, and chemical test. The mechanical parameters of the pelletization process, such as speed, angle, and duration, which were mentioned before, highly affect the mechanical properties of the cold-bonded aggregate (Manikandan and Ramamurthy 2007). Figure 3 illustrates the procedure for the many tests undertaken for this study. Consequently, the combination of these parameters helps us to produce a multi-granular size of the cold-bonded aggregate differing between 4 and 20 mm. The particle size distributions of three types of artificial green GCA are shown in Fig. 4. The characteristics were tabulated in Table 5.

**Table 5:** The physical and chemical characteristics of artificial green GCA

Sample name	Volume weight	Specific gravity	Crushing strength %	Absorption %	Chemical Cl and So_4_%
Mix (1)	1.26	2.22	24.80	3.09	0.009 and 0.13
Mix (2)	1.20	2.10	26.90	5.00	0.003 and 0.053
Mix (3)	1.08	1.90	23.10	9.00	0.043 and 0.064

**Fig. 5:**
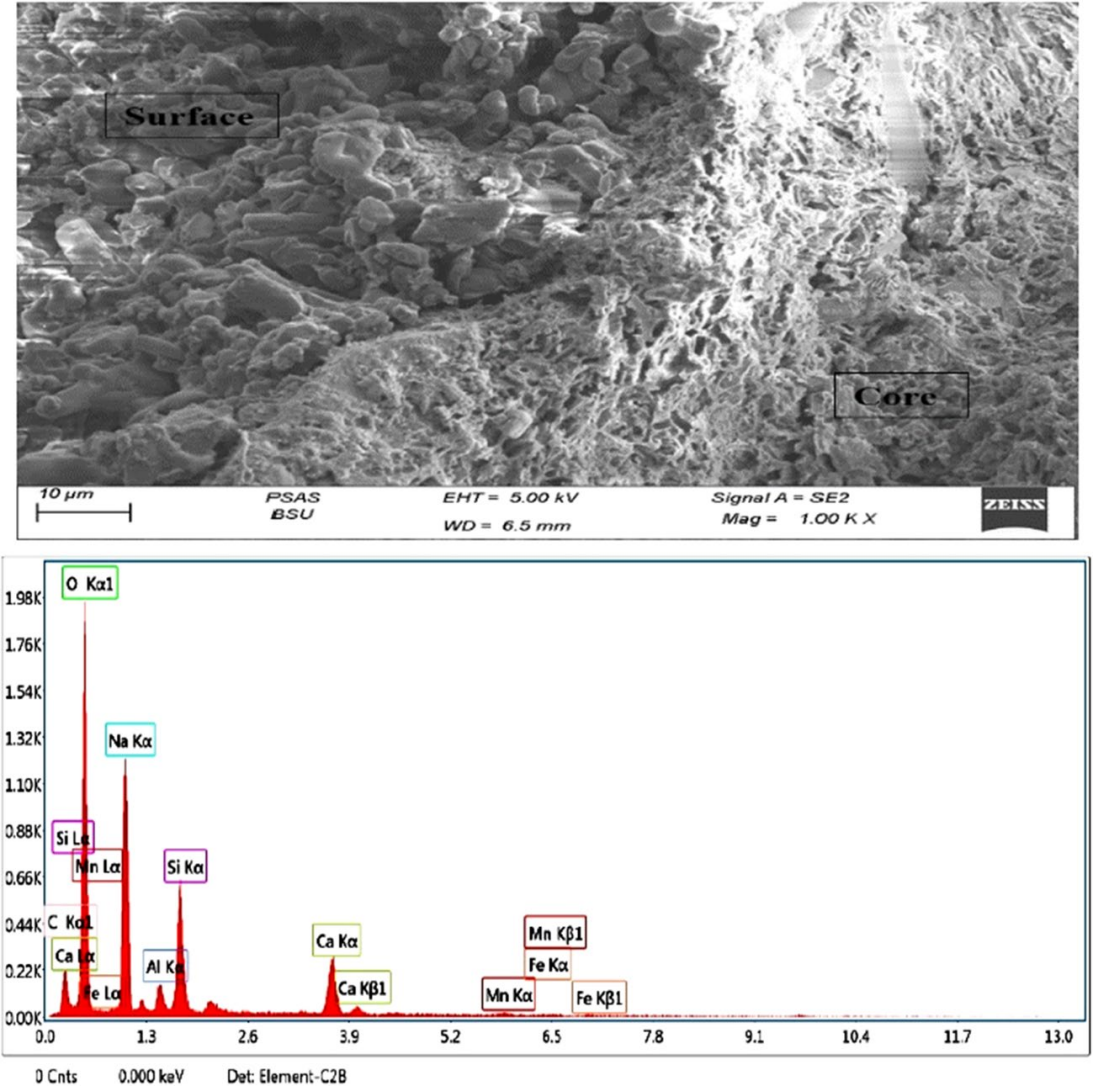
SEM and EDX analysis of mix (1) artificial green GCA

**Fig. 6:**
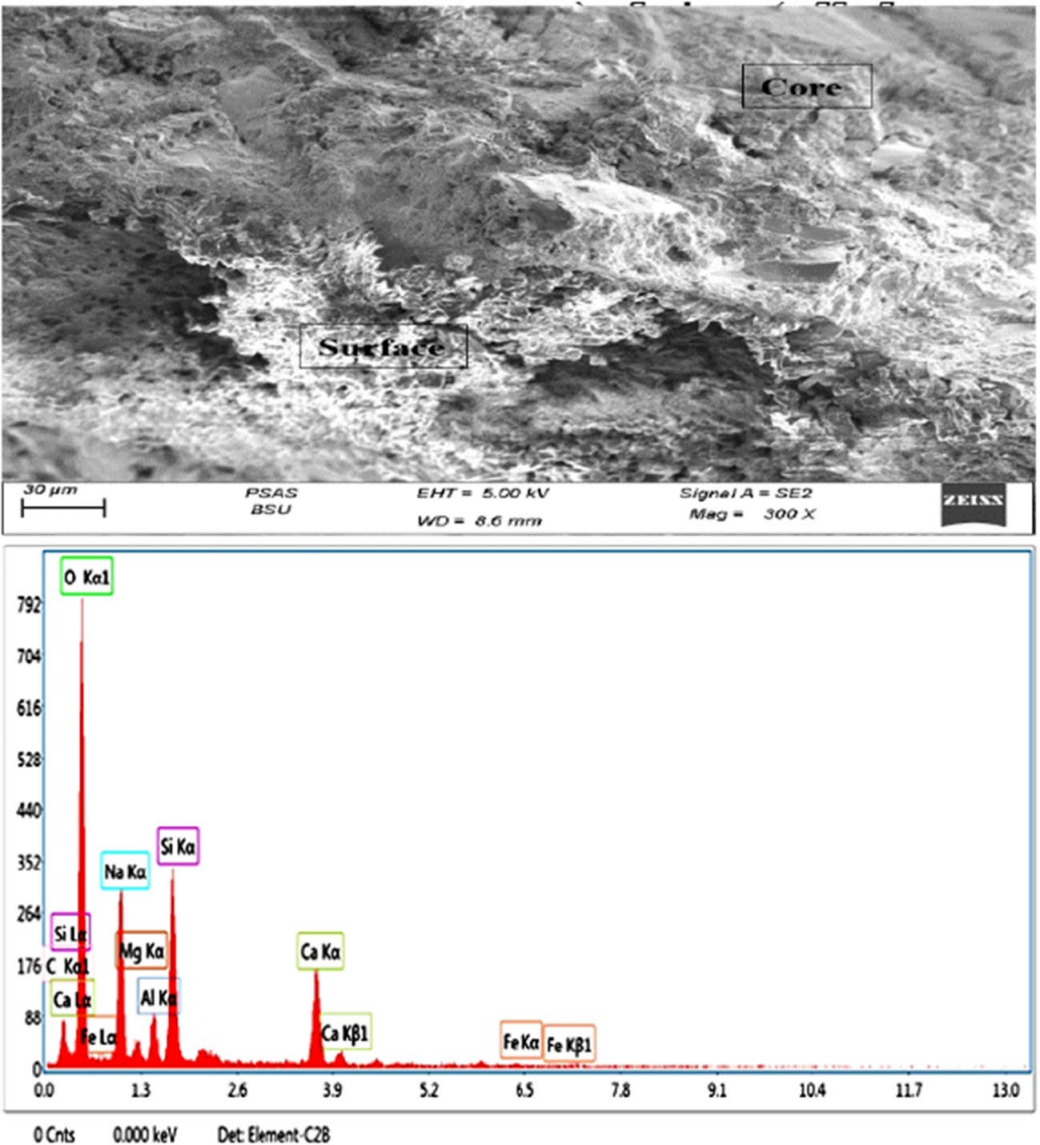
SEM and EDX analysis of mix (2) artificial green GCA

**Fig. 7:**
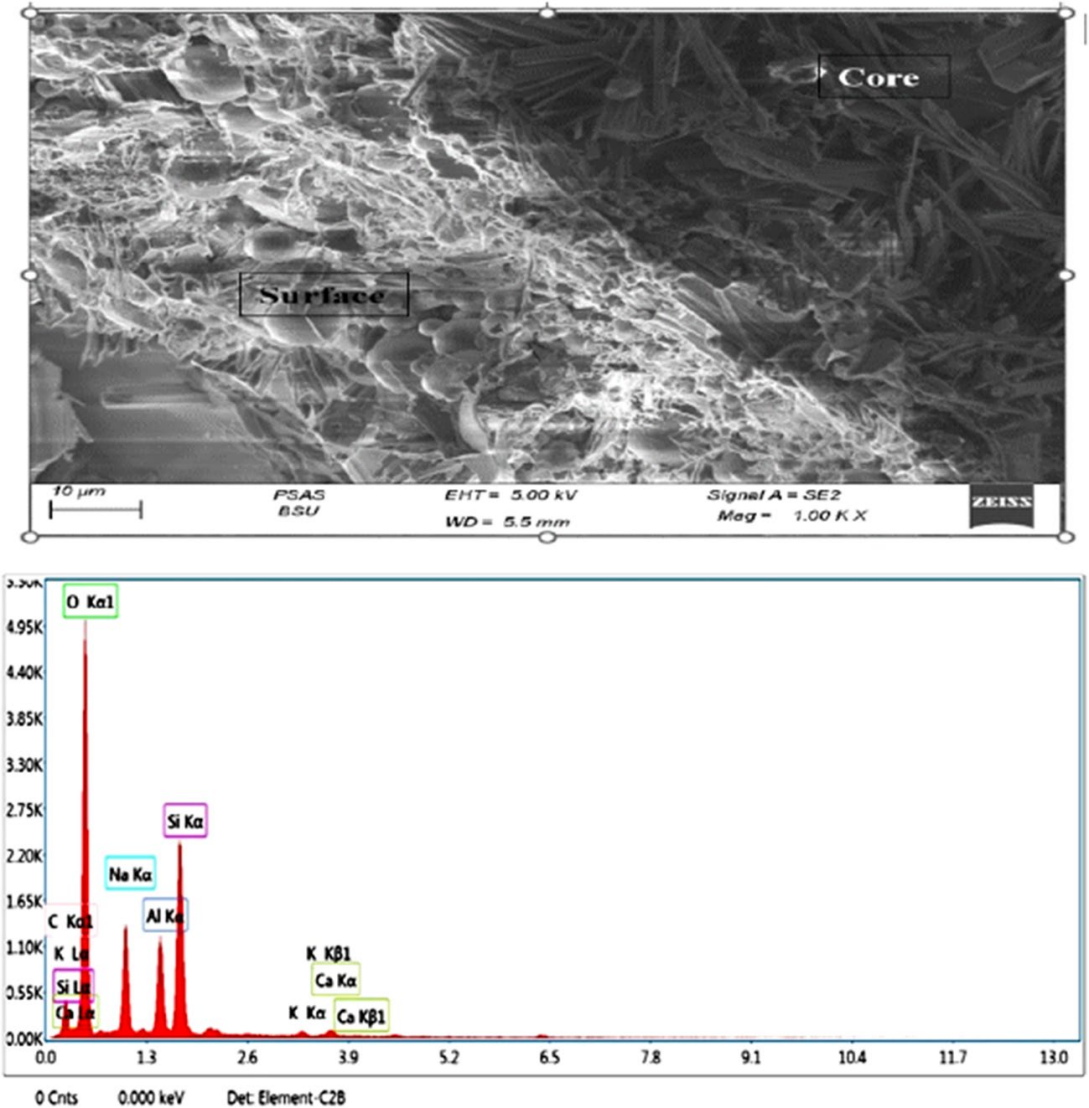
SEM and EDX analysis of mix (3) artificial green GCA

**Fig. 8:**
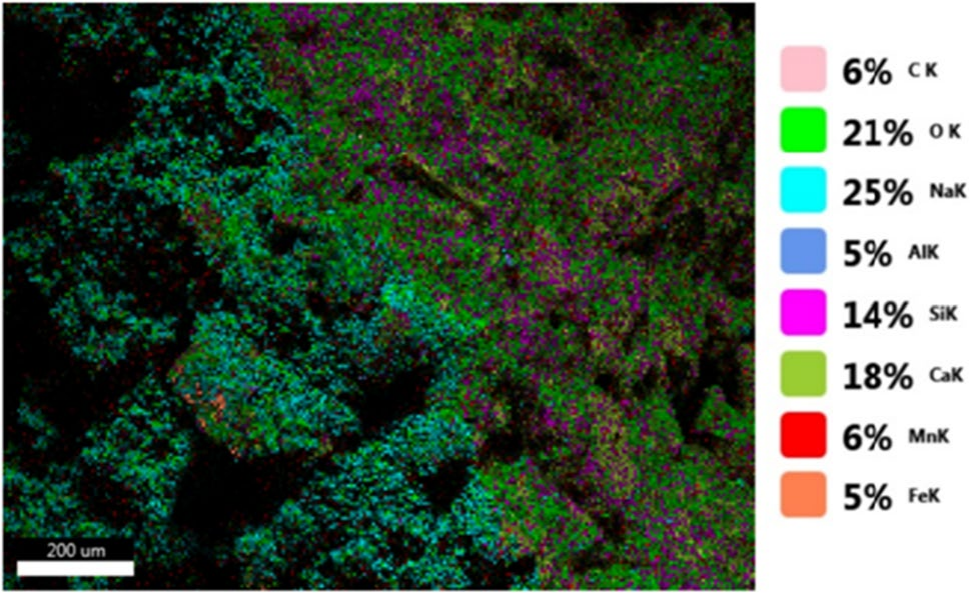
Mapping analysis of mix (1) artificial green GCA

In addition to physical and chemical properties, SEM analysis was used to get high-resolution images to check for surface cracks or defects in the artificial green GCA. Moreover, the elemental composition of a sample was determined quantitatively via EDX analysis, as shown in Figs. 5, 6, and 7. Also, the ingredients of the chemical members in the artificial green GCA were shown with the technology of EDX mapping, which was able to draw multiple colors, as displayed in Fig. 8. The characteristics are tabulated in Table 5.

### Concrete mixes and tests with GCA

According to Table 6, ten mix proportions were made by substituting natural aggregates with the artificial green geopolymer coarse aggregate (GCA) at percentages of 0%, 25%, 50%, and 75%. The extra water was added to the entire mixture because of the high absorption of the GCA. For each formulation, 10 × 10 × 10 cm^3^ cube-shaped specimens were cast. They were cured in water until the day of testing after demolding. The unit weights of hardened concrete were determined. At 7 days and 28 days, the compressive strength of the concrete specimens was tested and evaluated in accordance with Standard EN 12 390–3 (EN 12 390–3 2003).

**Table 6:** Concrete mix design

Series		NCA (Kg/m^3^)	GCA (Kg/m^3^)	Sand (Kg/m^3^)	Cement (Kg/m^3^)	Water (Kg/m^3^)	Extra water
**Control mix**		1292.78	0	695.92	350	157.5	**___**
**Group (1)**	M1-25%	969.58	323.2	695.92	350	157.5	**3.09% of ACA Wt**
	M1-50%	646.39	646.39	695.92	350	157.5	
	M1-75%	323.2	969.58	695.92	350	157.5	
**Group (2)**	M2-25%	969.58	323.2	695.92	350	157.5	**5.00% of ACA Wt**
	M2-50%	646.39	646.39	695.92	350	157.5	
	M2-75%	323.2	969.58	695.92	350	157.5	
**Group (3)**	M3-25%	969.58	323.2	695.92	350	157.5	**9.00% of ACA Wt**
	M3-50%	646.39	646.39	695.92	350	157.5	
	**M3-75%**	**323.2**	**969.58**	**695.92**	**350**	**157.5**	

*NCA*, natural coarse aggregate; *GCA*, artificial green geopolymer coarse aggregates

M1, M2 and M3 refers to (GGBFS), (GGBFS + perlite) and (Fly ash + perlite) respectively. (M1-25) 1 refers to the GCA mix containing GGBFS, and 25 refers to the GCA replacement ratio %. (M2-50) 2 refers to the GCA mix containing GGBFS + perlite, and 50 refers to the GCA replacement ratio%. (M3-75) 3 refers to GCA mix containing fly ash + perlite and 75 refer to GCA replacement ratio%

In order to assess the compressive strength at each of the indicated ages, three specimens were made from each mixture. At 28 days, the unit weight and water absorption of concrete were also measured. After the compressive test, numerous samples were analyzed for their microstructure using scanning electron microscopy (SEM). This operation was conducted to examine the interfacial transition zone (ITZ) between the cementitious matrix and the artificial green GCA.

Also cast and cured for 28 days were cylindrical samples of 150-mm diameter and 300-mm height made of concrete. The split tensile strength test was conducted using a 150-ton compression testing machine in accordance with ASTM C496-17 (ASTM C496 2017). The tests were conducted on specimens in triplicate, and the average values for split tensile strength were reported. In addition, the flexure strength test was performed in accordance with ASTM C293-16 (ASTM C293 2016) using 500 mm by 100 mm by 100 mm standard concrete beam that had been cured for 28 days. Using a universal testing machine with a 40-ton capacity and two-point loading, the test was conducted. The average flexural strength values of specimens tested in triplicate were determined.

## Results and discussion

### Unit weight

**Fig. 9:**
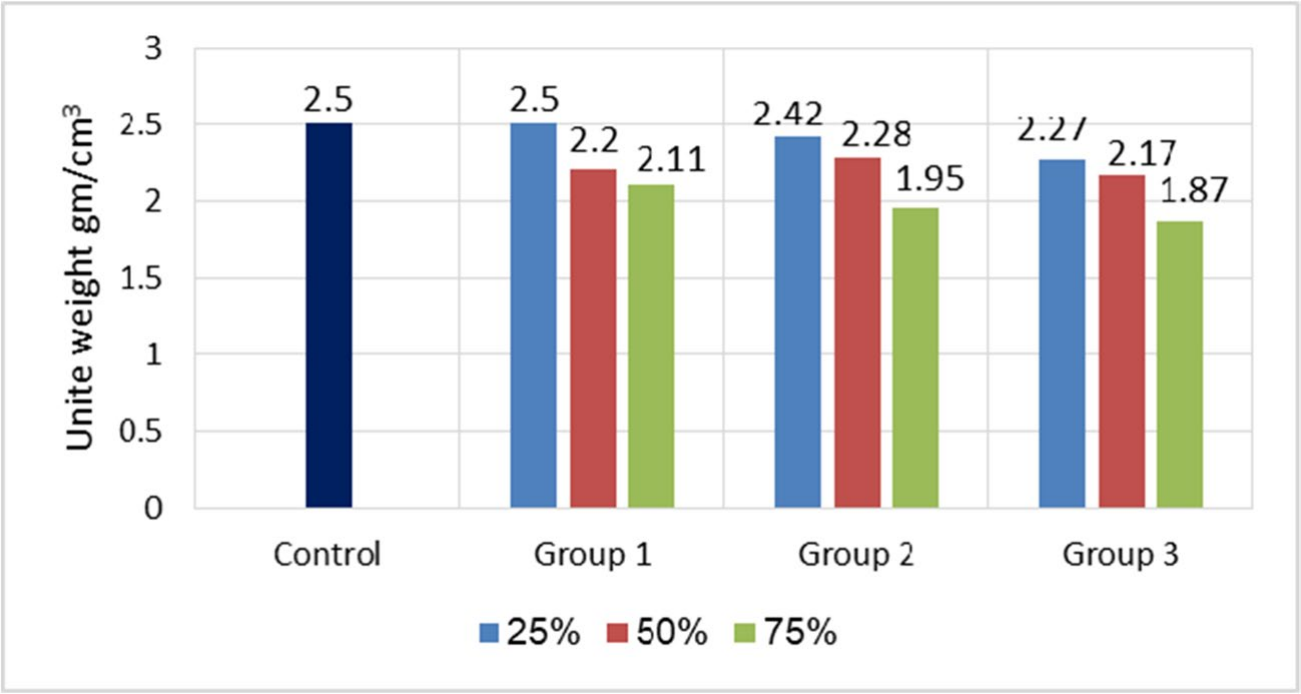
Unit weight comparison for all mixes used

The unit weight decreases gradually with increasing artificial green geopolymer coarse aggregate (GCA) content, as shown in Fig. 9. The specific weight of the natural aggregate (2.65) in the control sample was higher than that of the artificial green (GCA). The maximum value was noticed to be 2.5 gm/cm^3^ with 0% and 25% artificial green GCA in group one (M1), while the minimum value was 1.87 gm/cm^3^ with 75% artificial green (GCA) in group 3 (M3), and this was roughly 25% less compared to the reference concrete. Tang and Brouwers (2018) found that the density of normal aggregate concrete fell by approximately 5% and 8% when natural aggregate was substituted with cold-bonded aggregates at substitution levels of 30 vol% and 60 vol%, respectively. This research similarly observed the same pattern of decreasing density. The findings indicated that artificial green GCA group three (fly ash + perlite) results in a greater decline in unit weight compared to the control and group one (GGBFS) and group two (GGBFS + perlite) because the specific weight of fly is less than that of GGBFS. Another possible explanation might be that the specific gravity of the artificial green GCA (composed of fly ash and perlite) [1.9] is lower compared to the other groups [2.2 and 2.1]. As a result, the weight of the concrete dropped.

Furthermore, the value of M3-75% in group three was quite close to the upper limit of balanced density values ranging from 1120 to 1920 kg/m^3^ for structural lightweight aggregate concrete consisting of a mixture of lightweight and natural aggregate or entirely of lightweight aggregate, as specified by ASTMC 330 404 (ASTMC 330 404 2004). The findings of this study align with previous research (Bui et al. 2012). Given that the dry density of cold-bonded aggregate concrete was less than 2000 kg/m^3^, it is considered suitable for producing lightweight aggregate concrete, as specified in EN 206–1. This might potentially provide significant economic and environmental advantages by decreasing the overall weight of structures and enhancing their thermal efficiency.

### Compressive strength

**Fig. 10:**
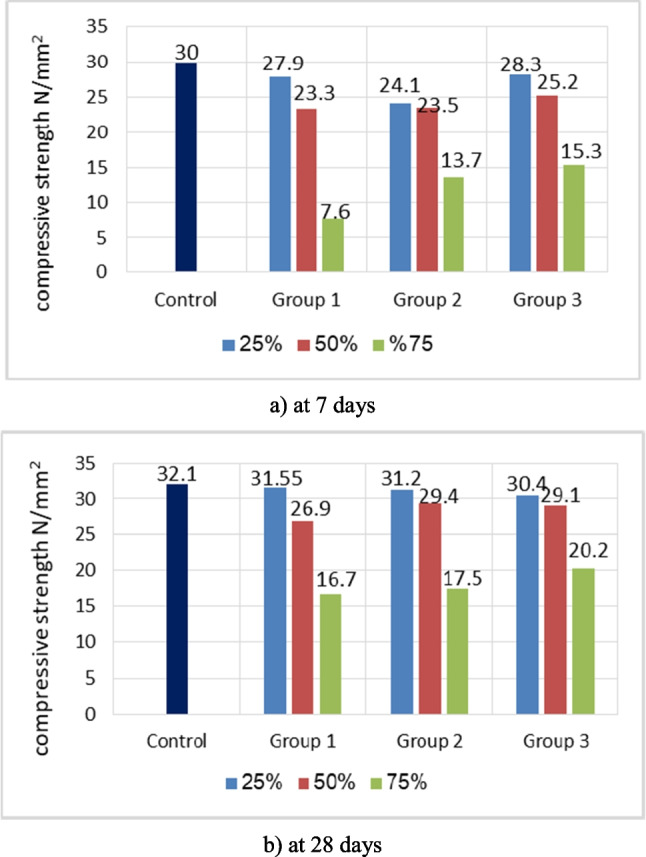
Compressive strength comparison for all mixes used at 7 days and 28 days

The evolution of compressive strengths of concrete evaluated at 7 days and 28 days as a function of different percentages of three types of artificial green geopolymer coarse aggregate(GCA) is presented graphically in Fig. 10.

Figure 10 clearly demonstrates that the compressive strength of concrete-artificial green GCA composites decreases as the substitution rate of artificial green GCA in the studied concrete increases. The compressive strength loss of M1-75%, M2-75%, and S M3-75% at 28 days was 47.9%, 45.5%, and 37.1% lower than that of the control concrete sample. The findings align with other studies (Gesogˆlu et al. 2014; Tang et al.^2017^), which documented a decrease in strength when cold-bonded aggregates were added to the concrete. The compressive strength of concrete was significantly influenced by the volume content of the aggregate (Yıldırım and Özturan 2013; Yang 1997). The other reason may be due to the artificial green GCA’s porosity, lesser hardness, and weakness compared to regular weight aggregate (İpek et al. 2020). In addition, the results of group three (fly ash + perlite) were closer to those of group one (GGBFS) and group two (GGBFS + perlite), which can be attributed to the physical characteristics of artificial green GCA particles, as the shape and particle size of artificial green GCA for group three were angular particles, whereas the shape of artificial green GCA particles in groups one and two were rounded. An additional explanation could be that the particle size of group three’s artificial green GCA was slightly lower than that of groups one and two. Due to its amorphous structure and higher surface area than fly ash, expanded perlite powder greatly enhanced the characteristics of cold-bonded aggregate concrete when used as a substitute for fly ash (Tajra et al. 2018). According to other studies, the performance of cold-bonded aggregate concrete can be improved by employing a binder with high reactivity. Besides, the current findings are in agreement with Bloem and Gaynor (1963), who discovered comparable aggregate size effects on the compressive strength of concrete. In addition, Nuruzzaman et al. (2020) observed that the angular form and rough texture of aggregates enhanced matrix-aggregate interface interlocking and adhesion.

On the other hand, it was observed that group two attained a lowered strength during the early curing age (7 days). At 28 days, however, it demonstrated a more rapid rate of strength development. This could be attributed to the denser interfacial transition zone (ITZ) in the former case, as the total number of micro cracks formed in the ITZ of concrete was comparatively lower at 28 days of age as opposed to 7 days. Additionally, the continual pozzolanic reaction within the hydrated cement matrix during later curing ages was thought to play a substantial role in contributing to the increase in strength (Gunasekera et al. 2018; Qian et al. 2020).

### Splitting tensile strength

**Fig. 11:**
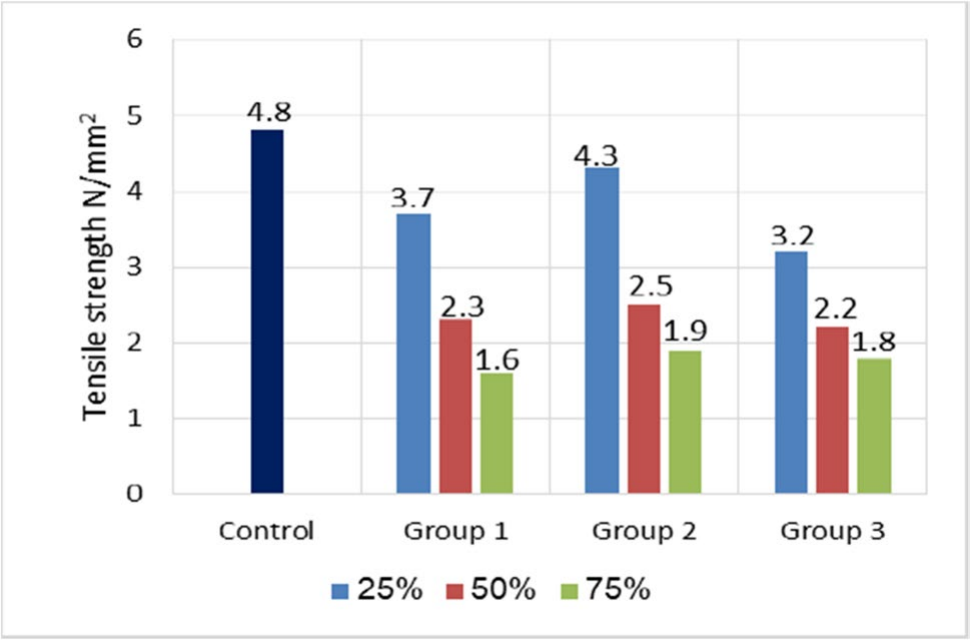
Splitting tensile strength comparison for all mixes used at 28 days

In Fig. 11, the 28-day splitting tensile strengths are displayed. The splitting tensile strength of the control concrete was determined to be 4.8 MPa, which fell by 22.9% to 66.7%, 10.4% to 60.4%, and 33.3% to 62.5% for combinations in groups one (GGBFS), two (GGBFS + perlite), and three (fly ash + perlite), respectively. Similar to the trend observed with compressive strengths, splitting tensile strength dropped as the content of various types of artificial green geopolymer coarse aggregate (GCA) increased in comparison with the control sample. Thus, the splitting tensile strength of concrete made with artificial green GCA correlated well with its compressive strength, as is normally found in conventional concrete made with natural aggregates. The substitution of normal aggregate with cold-bonded aggregates was found to have a detrimental effect on the splitting strength of concrete, as cold-bonded aggregates have a lower strength than normal aggregate (Yıldırım and Ozturan 2013). In a study conducted Gesogˆlu et al. (2014), it was observed that cold-bonded aggregate concrete containing 100% cold-bonded aggregates exhibited a 44% reduction in tensile strength when compared to normal aggregate concrete.

On the other hand, it was observed that group two exhibited a greater splitting tensile strength when compared to both group one and group three. This disparity may be attributed to the presence of a more dense interfacial transition zone (ITZ). The results coincide with previous research concerning the fact that tensile strength is more related to the cementitious matrix and especially to the adhesion between the concrete constituents (Saidaniet al.^2015^).

Compared to natural coarse aggregate, manufactured green GCA had a lower tensile strength due to its low density, weakness, and porous structure. This could potentially be due to insufficient adhesion between the cement matrix and artificial green GCA particles (as discussed in SEM analysis).

### Flexural strength

**Fig. 12:**
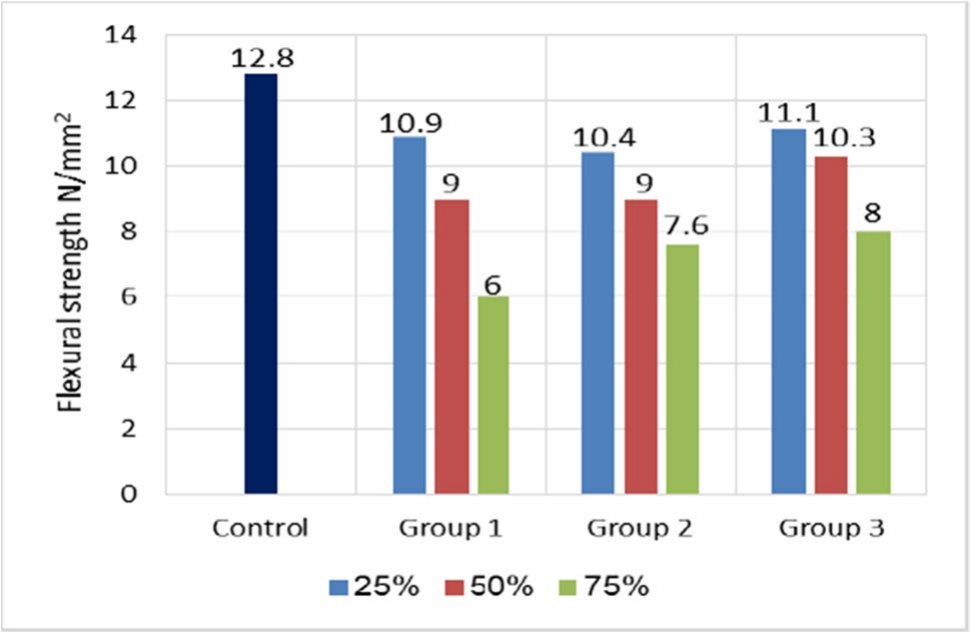
Flexural strength comparison for all mixes used at 28 days

Figure 12 illustrates the flexural strength of concrete specimens prepared with varying mixture amounts. Obviously, the flexural strength decreased as the ratio of artificial green geopolymer coarse aggregate increased (GCA). However, the flexural strength values decreased from 12.8 MPa at the 0% reference mixture to 6 MPa, 7.6 MPa, and 8 MPa at 75% of GGBFS, 75% of GGBFS + perlite, and 75% of fly ash + perlite, respectively. Similarly, Terzic´et al. (2015) reported that cold-bonded aggregate concrete containing 100% cold-bonded aggregates exhibited a flexural strength 40–47% lower than normal aggregate concrete. Kumar et al. (2014) further determined that the percentage loss in flexural strength for cold-bonded aggregate concrete was 29%. In this test, a significant distinction was noticed between concrete made with artificial green GCA and concrete made with natural aggregates. The artificial green GCA exhibited the lowest flexural strength especially when increasing replacement percentage.

This is primarily due to the fact that the artificial green GCA particles were weaker. Due to the rounded shape of the particles, particularly in groups one and two, these aggregates have a decreased capacity for anchorage with solidified cement paste (Li et al. 2000). This was a significant drawback. It was recognized that such aggregate has a lower crushing strength than natural aggregate. When the mechanism of flexural strength was explored, it was clear that aggregate plays a key role in this behavior. Because the crack caused by the applied force on the specimen seeks weaker propagation routes, utilizing a weaker aggregate will result in lower splitting tensile behavior due to the ease of crack formation and propagation in the event of using artificial green GCA (Sarhatand Sherwood; Rezaei Lori et al. 2019).

### Water absorption

**Fig. 13:**
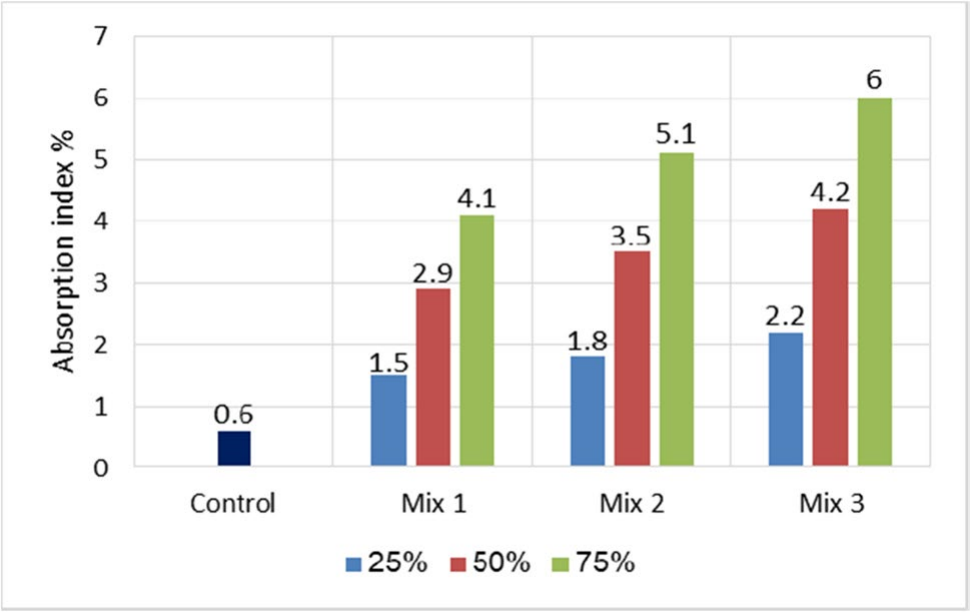
Water absorption comparison for all mixes used at 28 days

This is considered one of the most important durability qualities of concrete, which infers the water accessibility of its pore network. Figure 13 depicts the results of the water absorption test. The obtained results indicated that water absorption increases as the substitution rate of artificial green geopolymer coarse aggregate (GCA) particles with various combinations increases; 75% of fly ash + perlite concrete exhibited the optimal increase in water absorption relative to the control concrete. In fact, after 24 h of immersion in water, the rate of increase in water absorption was 4.1%, 3.5%, and 6% for mortars containing 75% GGBFS, 75% GGBFS + perlite, and 75% fly ash + perlite, respectively.

It is fascinating to consider that this improvement in absorption is likely attributable to the hydrophilic nature of artificial green GCA particles. Previous research (Sathiparan and De Zoysa 2018) attributed this increase in absorption to the fact that cementitious matrix often absorbs a great deal of water as a result of the matrix’s increased porosity. As per the findings of Balapour et al. (2020), it was noticed that cold-bonded aggregate exhibited quick absorption within the initial few hours. This can be attributed to the capillary suction of empty capillary holes, which were typically smaller than 10 μm in size. Subsequently, the rate at which water was absorbed decreased, indicating the secondary absorption associated with bigger pores. Thus, in the current research, group three contains a huge number of large pores, which could explain the reason for high water absorption compared to other groups. Although group three recorded the highest water absorption, it had a positive impact on compressive strength at 7 days and 28 days. Obviously, the compressive strength was higher at 7 days compared to other groups. At the same instant, group three conserved its compressive strength. At the early stage, the concrete shrinkage phenomenon is mitigated through the release of water absorbed by GCA, and consequently, the concrete shrinkage is dependent on the water absorption capacity of artificial green GCA.

### Relationship between the tested properties

**Fig. 14:**
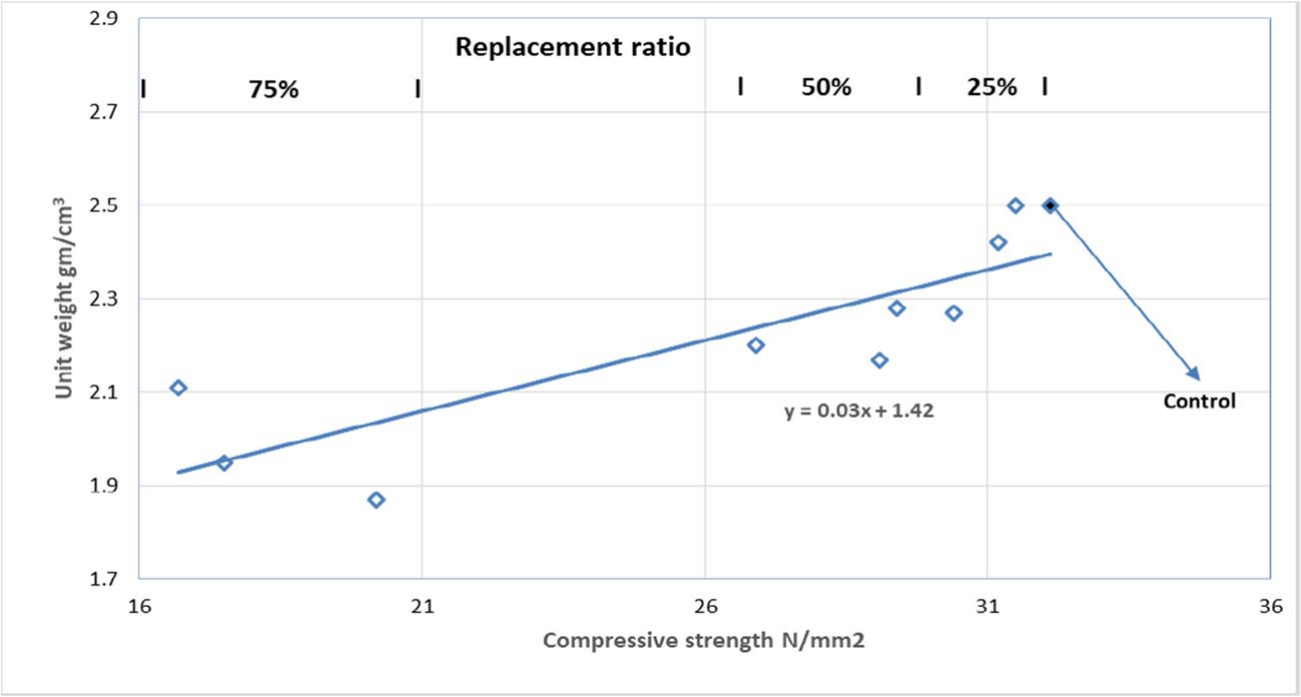
Unit weight and compressive strength

Figure 14 illustrates the relation between the compressive strength and unit weight at different ratios of replacement. The higher the replacement ratio, the lower the unit weight of the concrete produced and the lower the compressive strength of the concrete produced, resulting in the decreased crushing strength of the produced artificial coarse aggregate.

**Fig. 15:**
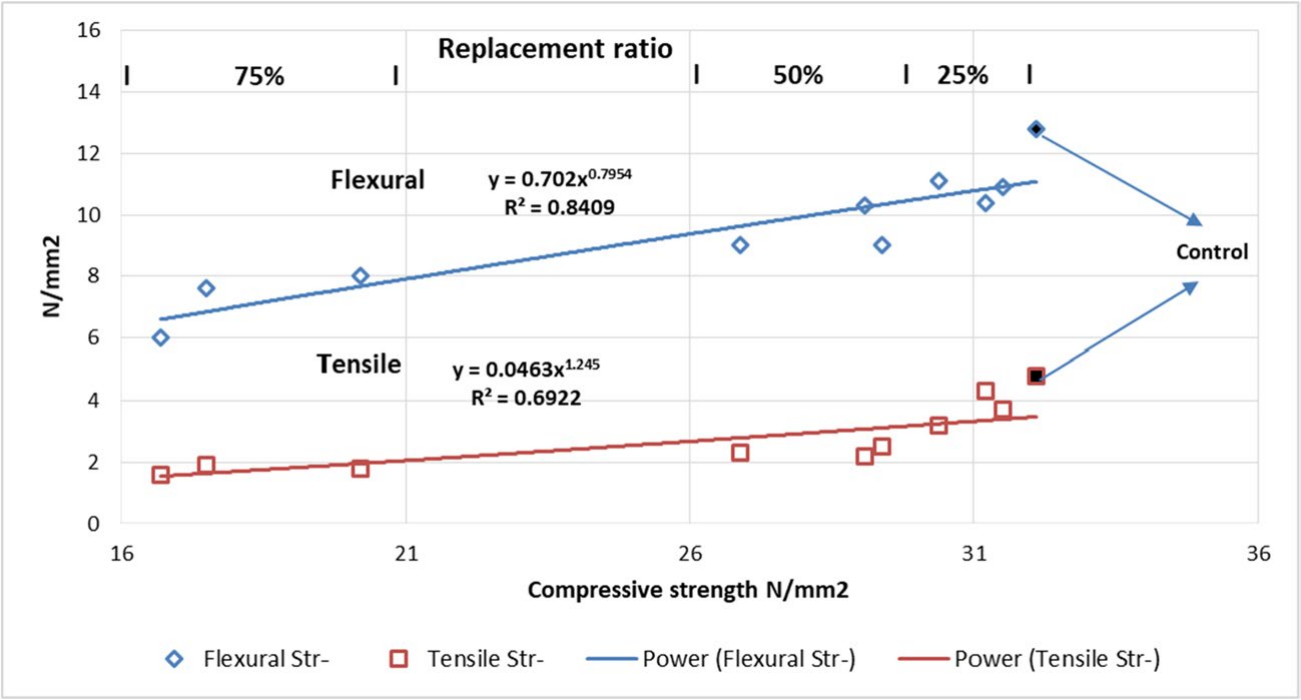
Flexural strength and compressive strength

Figure 15 illustrates the relationship between compressive strength, flexural strength, and tensile strength. At different ratios of replacement, the higher the replacement ratio, the lower the flexural and tensile strength of the produced concrete, resulting in a decrease in unit weight and crushing strength of the produced coarse aggregate.

**Fig. 16:**
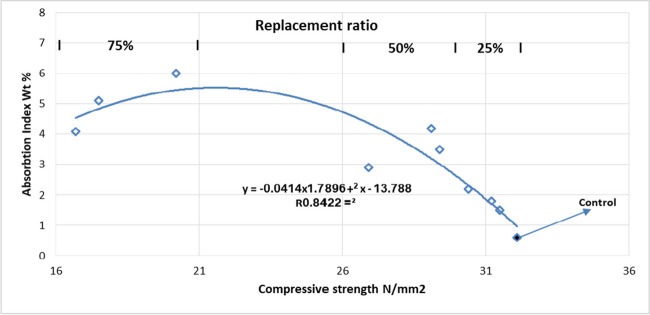
Water absorption and compressive strength

Also, Fig. 16 illustrates the relationship between compressive strength and absorption index at different ratios of replacement. The higher the replacement ratio, the higher the absorption index of the produced concrete, resulting in pores and voids in the core of the produced coarse aggregate, besides the high absorption index of perlite and slag.

### Microstructure of concrete

Concrete consists of the cement matrix, aggregate, and interfacial transition zone between them; it is known as the ITZ, which influences the durability and mechanical properties of the concrete. Therefore, in normal concrete, and due to the large differences between aggregate and cement particles, a weak area is created on the surface of the aggregate. This area contains a large amount of water and fewer cement particles, which produce a weak ITZ, leading to a reduced bond between aggregate and cement paste (Zhang and Gjørv 1990).

**Fig. 17:**
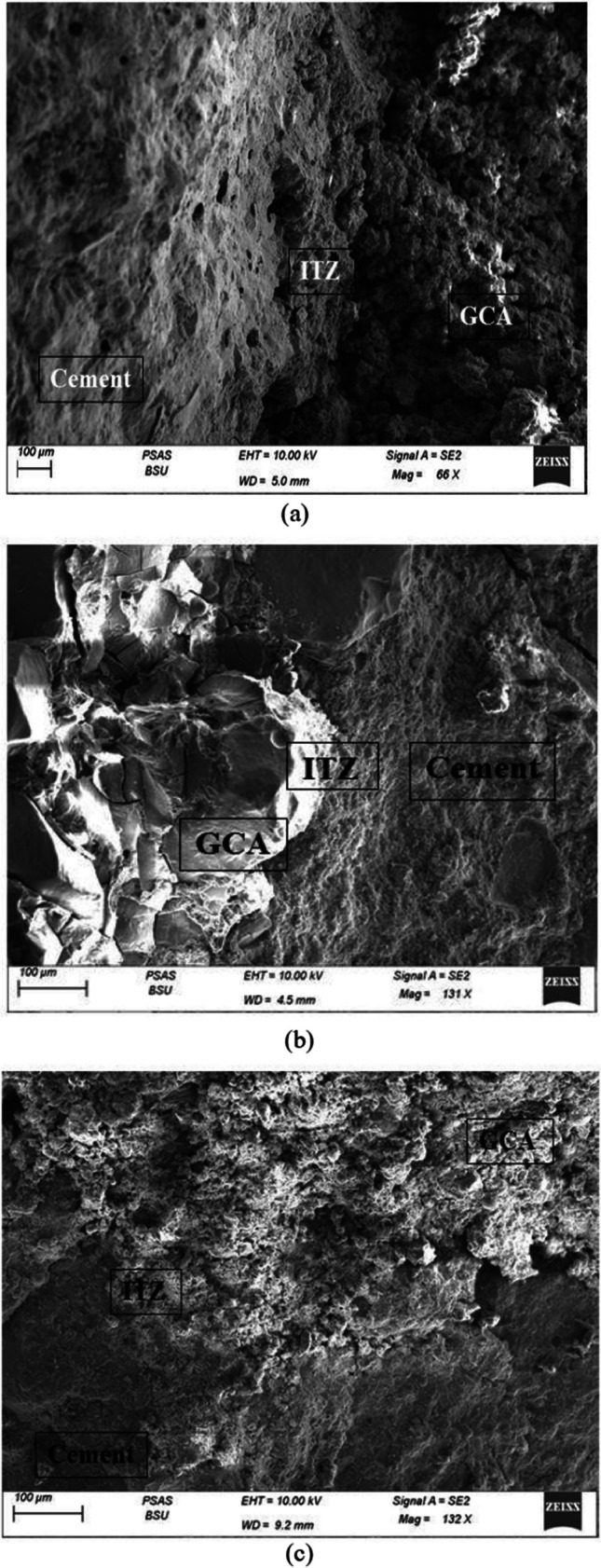
SEM images of (**a**) 50% of GGBFS, (**b**) 50% of GGBFS + perlite, and (**c**)50% of fly ash + perlite, respectively

Figure 17 illustrates that ITZ almost overlaps with the artificial aggregate surface because of the pores and the sinuosity in the surface of the aggregate, which is mainly formed by the reaction between sodium hydroxide and sodium silicate in the cold-bonded process. Besides, the cement hydration process produces calcium hydroxide, which could interact with the raw material that was used in producing aggregate. Furthermore, it is obvious that the morphology of the 50% GGBFS + perlite mix is a bit more dense and compacted when compared to the other mixes, as seen in Fig. 17b. Nevertheless, the microstructure of 50% of fly ash + perlite (Fig. 17c) exhibited a minor porous nature, with more distinct grains indicating less reactivity as compared to the 50% of GGBFS and 50% of GGBFS + perlite specimens (Fig. 17a,b). This result aligns with the strength finding, as the water absorption of 50% of the mixture of fly ash and perlite exhibited the greatest value.

The failure of the concrete under compression seemed to occur away from the ITZ between the artificial aggregate and the cement paste, either in the core of the artificial aggregate or between the ITZ of natural aggregate. According to Fig. 17, the cracks appear clearly in the sample that was chosen after failure under a compressive test machine.

## Environmental impact

Ground-granulated blast furnace slag (GGBFS), silica fume, and fly ash, and specifically, the GGBFS and fly ash wastes utilized in current research, are all byproducts of iron and steel production, coal-fired electric and steam generating plants, and coal-fired electric power plants, respectively. According to Hossain et al. (2016), 1 tonne of natural coarse aggregates from crushed stone releases approximately 32 kgCO_2_eq of greenhouse gasses (GHGs). Table 7 shows the carbon emission of artificial green geopolymer coarse aggregates (GCA).

**Table 7:** Carbon emission produced by the artificial green GCA

Artificial green geopolymer coarse aggregates (GCA)	Mass of pelletized (t)	Pelletization time (h)	Oven time for 24 h at 80 °C (h)	Power (kW)	Electricity consumption (kWh/ t)	CO_2_ emission of electricity (kg/t)
GCA for concrete mixer	0.3	0.05	-	0.5	0.083	0.015
GCA for oven	0.3	-	24	0.5	40	7.4

Note: the CO_2_ emission of electricity by burning natural gas is 0.185 kg/kWh, according to DEFRA (2007)

The CO_2_ emission reduction extent, Ψ, can be calculated by

$$\Psi =\left(\frac{GH{G}_{GCA}}{GH{H}_{NA}}\right)\times n\mathrm{\%}$$

By substituting GHG_GNA_ = 32 kWh/t and GHG_GCA_ = 7.415 kWh/t into the previous equation yields to Ψ = 0.768 × *n*%. This indicates that every 25%, 50%, and 75% of NA substituted by artificial green GCA can reduce total CO_2_ emissions by 19.2%, 38.4%, and 57.6%. The use of waste materials (GGBF and FA) in the production of GCA results in reduced emissions of CO_2_-equivalent. GCA is a promising environmentally friendly material that can be employed in many engineering applications. Therefore, GCA made with GGBF, GGBF + perlite, and fly ash + perlite exhibited the most favorable environmental characteristics. This suggests that using industrial byproducts and construction waste for the production of GCA is an efficient strategy to accelerate the attainment of carbon peak and carbon neutral objectives. However, the ultimate choice of materials must thoroughly take into account their characteristics and carbon dioxide emissions.

## Conclusion

This study illustrated producing artificial green gepolymer coarse aggregates (GCA) using waste materials such as GGBFS and fly ash with new cold-bonded technology. Besides, we studied the effect of using GCA as a partial replacement of natural coarse aggregate on the mechanical and durability properties of concrete. Also, we studied how far this impact on the behavior of concrete mechanical properties and its durability. In addition, SEM evaluations of the microstructural behavior of concrete were conducted. The following are the comprehensive conclusions based on the findings of the investigation.

- Producing artificial green GCA with the traditional lab mixer is a suitable, economical, and easy technique with slag and fly ash as a base material besides being an eco-friendly recycling method and could not be just a waste material.
- Artificial aggregate has a round shape and sinuosity surface, which leads to high workability and consumes less cement content for bonding.
- The mechanical properties of GCA produced by the cold-bonded technique are similar to the natural aggregate, with the additional benefit of using recycled waste materials such as GGBFS and fly ash. However, the absorption of the GCA was higher than the natural aggregate.
- Generally, the compressive strength, splitting strength, flexural strength, and unit weight of GCA were decreased by increasing the artificial aggregate content. Nevertheless, the water absorption of concrete for all mixtures was increased by increasing GCA content.
- Lightweight concrete was created when a 75% replacement ratio of GCA was used with mix 3 (fly ash + perlite), while its semi-lightweight concrete was obtained with 75% GCA in mix 2 (GGBFS + perlite) that could be used for non-structural purposes. In addition, this was considered a beneficial option for addressing the energy balance issues in the building industry. By adopting this approach, the thermal efficiency of the building may be greatly enhanced.
- The maximum compressive strength in comparison with other groups with GCA at 28 days was at a 25% replacement ratio of group 1 (GGBFS) equal to 31.55 N/mm^2^, decreasing by 1.7% compared to the control mix as a result of a high density of ACA and low crushing strength.
- The maximum splitting strength in comparison with other groups was at a 25% replacement ratio of mix 2 (GGBFS + perlite) equal to 4.3 N/mm^2^, decreasing by 10.4% compared to the control mix.
- The maximum flexural strength in comparison with other groups was at a 25% replacement ratio of mix 3 (fly ash + perlite) equal to 11.1 N/mm^2^, decreasing by 13.3% compared to the control mix.
- The maximum water absorption in comparison with other groups was at a 75% replacement ratio of mix 3 (fly ash + perlite), equal to 6%, increasing by 900% compared to the control mix.
- SEMs showed a good ITZ which was created with GCA and cement paste due to overlapping between aggregate surface and cement particles.
- The failure of the compressive strength test occurred away from the ITZ between artificial aggregate and cement paste, according to SEM.
- This study demonstrated a manufacturing technique that could enhance the practical application of cold-bonded GCA, thereby causing a reduction in the energy consumption and pollution caused by the sintering technique, which was illustrated in the environmental impact. Moreover, this new technique may encourage the use of a wide variety of waste materials in the production of GCA.

## Data Availability

Not applicable.

## References

[CR1] Agrawal US, Wanjari SP, Naresh DN (2019) Impact of replacement of natural river sand with geopolymer fly ash sand on hardened properties of concrete. Constr Build Mater 209:499–507

[CR2] Ahmed HU, Mahmood LJ, Muhammad MA, Faraj RH, Qaidi SM, Sor NH, Mohammed AS and Mohammed AA (2022) Geopolymer concrete as a cleaner construction material: an overview on materials and structural performances. Cleaner Materials 5:00111

[CR3] Ali N, Jaffar A, Anwer M, Khan S, Anjum M, Hussain A, Raja M and Ming X (2015) The greenhouse gas emissions produced by cement production and its impact on environment: a review of global cement processing. Intl J Res (IJR) 2(2)

[CR4] Aljerf L (2015). Effect of thermal-cured hydraulic cement admixtures on the mechanical properties of concrete. Interceram - Intl Ceram Rev.

[CR5] Alrefaei Y, Wang YS, Dai JG (2019). The effectiveness of different superplasticizers in ambient cured one-part alkali activated pastes. Cem Concr Compos.

[CR6] ASTM C293/C293M-16, Standard test method for flexural strength of concrete (using simple beam with center-point loading), ASTM International, West Conshohocken, PA, 2016.

[CR7] ASTM C496/C496M-17, Standard test method for splitting tensile strength of cylindrical concrete specimens, ASTM International, West Conshohocken, PA, 2017.

[CR8] Balapour M, Zhao W, Garboczi EJ, Oo NY, Spatari S, Hsuan YG, Billen P, Farnam Y (2020) Potential use of lightweight aggregate (LWA) produced from bottom coal ash for internal curing of concrete systems. Cem Concr Compos 105:103428

[CR9] Bijen J (1986). Manufacturing processes of artificial lightweight aggregates from fly ash. Intl J Cement Compos Lightweight Concrete.

[CR10] Bloem DL, Gaynor RD (1963). Effects of aggregate properties on strength of concrete. J Proc.

[CR11] Bui LAT, Hwang CL, Chen CT, Lin KL, Hsieh MY (2012). Manufacture and performance of cold bonded lightweight aggregate using alkaline activators for high performance concrete. Constr Build Mater.

[CR12] Cao Y, Wang Y, Zhang Z, Wang H (2022). Recycled sand from sandstone waste: a new source of high-quality fine aggregate. Resour Conserv Recycl.

[CR13] Colangelo F, Cioffi R (2013). Use of cement kiln dust, blast furnace slag and marble sludge in the manufacture of sustainable artificial aggregates by means of cold bonding pelletization. Materials.

[CR14] DEFRA (2007) Guidelines to Defra's GHG conversion factors for company reporting www.defra.gov.uk or www.carbonindependent.org/files/conversion-factors.pdf

[CR15] Gomathi P, Sivakumar A (2015) Accelerated curing effects on the mechanical performance of cold bonded and sintered fly ash aggregate concrete. Construction Build Mater 77:276–287

[CR16] Geetha S, Ramamurthy K (2013). Properties of geopolymerised low-calcium bottom ash aggregate cured at ambient temperature. Cement Concr Compos.

[CR17] George GK, Revathi P (2020) Production and utilisation of artificial coarse aggregate in concrete-a review. In IOP Conf Ser: Mater Sci Eng 936(1):012035. IOP Publishing

[CR18] Gesogˆlu M, Güneyisi E, Özturan T, Öz HÖ, Asaad DS (2014). Permeation characteristics of self compacting concrete made with partially substitution of natural aggregates with rounded lightweight aggregates. Constr Build Mater.

[CR19] Gunasekera C, Law D, Setunge S (2018). Effect of geopolymer aggregate on strength and microstructure of concrete. ACI Mater J.

[CR20] Hossain MdUzzal, Poon Chi Sun, Lo Irene MC, Cheng Jack CP (2016). Comparative environmental evaluation of aggregate production from recycled waste materials and virgin sources by LCA. Resour Conserv Recycl.

[CR21] Hwang CL, Tran VA (2015) A study of the properties of foamed lightweight aggregate for self-consolidating concrete. Constr Build Mater 87:78–85

[CR22] İpek S, Ayodele OA, Mermerdaş K (2020). Influence of artificial aggregate on mechanical properties, fracture parameters and bond strength of concretes. Constr Build Mater.

[CR23] Kumar VRP, Anandh KS, Kumar M (2014). An experimental study on partial replacement of natural coarse aggregate with fly ash coarse aggregate (FACA). Res Appl Sci Eng Technol.

[CR24] Li Y, Wu D, Zhang J, Chang L, Wu D, Fang Z, Shi Y (2000). Measurement and statistics of single pellet mechanical strength of differently shaped catalysts. Powder Technol.

[CR25] Manikandan R, Ramamurthy K (2007). Influence of fineness of fly ash on the aggregate pelletization process. Cement Concr Compos.

[CR26] Manikandan R, Ramamurthy K (2009). Swelling characteristic of bentonite on pelletization and properties of fly ash aggregates. J Mater Civ Eng.

[CR27] Mankelow JM, Oyo-Ita D, Birkin M, 2010 Assessing the carbon footprint of transporting primary aggregates. In: Scott PW, Walton G (Eds.), Proceedingsof the 15th Extractive Industry Geology Conference. EIG Conferences Ltd, pp.41–45 (p186).

[CR28] Minh DQ, Thang NH, 2020 Characteristics of novel geopolymer composites synthesized from red mud and diatomaceous earth in autoclave conditions without using alkaline activators. J Polym Compos 8 (3).

[CR29] Naik TR (2008). Sustainability of concrete construction. Pract Period Struct Des Constr.

[CR30] Nandy S, Fortunato E, Martins R (2022) Green economy and waste management: an inevitable plan for materials science. Progr Nat Sci Mater Intl 32(1):1–9

[CR31] Nuruzzaman MD, Casimiro JOC, Sarker PK (2020). Fresh and hardened properties of high strength self-compacting concrete using by-product ferronickel slag fine aggregate. J Build Eng.

[CR32] Provis JL, Palomo A, Shi C (2015). Advances in understanding alkali-activated materials. Cem Concr Res.

[CR33] Qian LP, Wang YS, Alrefaei Y, Dai JG (2020). Experimental study on full-volume fly ash geopolymer mortars: sintered fly ash versus sand as fine aggregates. J Cleaner Prod.

[CR34] Rehman MunibUl, Rashid Khuram, EhsanUlHaq MuridHussain, NasirShehzad.,  (2020). Physico-mechanical performance and durability of artificial lightweight aggregates synthesized by cementing and geopolymerization. Const Build Mater.

[CR35] Rezaei Lori A, Hassani A, Sedghi R (2019). Investigating the mechanical and hydraulic characteristics of pervious concrete containing copper slag as coarse aggregate. Construct Build Mater.

[CR36] Risdanareni P, Ekaputri JJ, Maulidiyawati I, Puspitasari P (2018) Mechanical properties of concrete composed of sintered fly ash lightweight aggregate. In MATEC Web of Conferences (Vol 95, p 01008). EDP Sciences

[CR37] Saidani K, Ajam L, Ouezdou MB (2015). Barite powder as sand substitution in concrete: effect on some mechanical properties. Constr Build Mater.

[CR38] Sathiparan N, De Zoysa HTSM (2018). The effects of using agricultural waste as partial substitute for sand in cement blocks. J Build Eng.

[CR39] Scrivener KL, Crumbie AK, Laugesen P (2004) The interfacial transition zone (ITZ) between cement paste and aggregate in concrete. Interface Sci 12:411–421

[CR40] Shilar FA, Ganachari SV, Patil VB, Khan TY, Dawood S (2022) Molarity activity effect on mechanical and microstructure properties of geopolymer concrete: a review. Case Stud Construct Mater 16:e01014

[CR41] Sims I, and Brown B, “Concrete aggregates,” in Lea’s Chemistry of Cement and Concrete, Elsevier, 1998, pp. 907–1015 10.1016/B978-075066256-7/50028-X.

[CR42] Sivakumar A, Gomathi P (2012). Pelletized fly ash lightweight aggregate concrete: a promising material. J Civil Eng Construct Technol.

[CR43] Tajra F, AbdElrahman M, Chung SY, Stephan D (2018). Performance assessment of core-shell structured lightweight aggregate produced by cold bonding pelletization process. Constr Build Mater.

[CR44] Tajra F, AbdElrahman M, Stephan D (2019). The production and properties of cold-bonded aggregate and its applications in concrete: a review. Constr Build Mater.

[CR45] Tang P, Brouwers HJH (2018). The durability and environmental properties of selfcompacting concrete incorporating cold bonded lightweight aggregates produced from combined industrial solid wastes. Constr Build Mater.

[CR46] Tang P, Florea MVA, Brouwers HJH (2017). Employing cold bonded pelletization to produce lightweight aggregates from incineration fine bottom ash. J Cleaner Prod.

[CR47] Terzic´ A, Pezo L, Mitic´ V, Radojevic Z (2015). Artificial fly ash based aggregates properties influence on lightweight concrete performances. Ceram Int.

[CR48] Thang NH, 2020 Geopolymerization: a review on physico-chemical factors influence to the reaction process. J Polym Compos 8 (3).

[CR49] Wang Q, Ko JH, Liu F, Xu Q (2021) Leaching characteristics of heavy metals in MSW and bottom ash codisposal landfills. J HazardMater 416:126042 10.1016/j.jhazmat.2021.126042 34492889

[CR50] Yang CC (1997) Approximate elastic moduli of lightweight aggregate. Cement and concrete research 27(7):1021–1030

[CR51] Yıldırım H, Özturan T (2013) Mechanical properties of lightweight concrete made with cold bonded fly ash pellets

[CR52] Zhang MH, Gjørv OE (1990). Microstructure of the interfacial zone between lightweight aggregate and cement paste. Cem Concr Res.

